# A reflection on ketoABNO: the crossing point between organic synthesis and protein modification

**DOI:** 10.1039/d5sc90203g

**Published:** 2025-10-02

**Authors:** Moe Toyobe, Motomu Kanai

**Affiliations:** a Graduate School of Pharmaceutical Sciences, The University of Tokyo Japan kanai@mol.f.u-tokyo.ac.jp

## Abstract

In 2012, we reported that the *N*-oxyl radical ketoABNO functions as an effective catalyst for the mild aerobic oxidation of amines to imines (T. Sonobe, K. Oisaki and M. Kanai, *Chem. Sci.*, 2012, **3**, 3249, https://doi.org/10.1039/C2SC20699D). Its catalytic versatility arises from a unique combination of steric compactness, high oxidation potential, and the ability to reversibly interconvert among three oxidation states—hydroxyamine, *N*-oxyl, and oxoammonium. Beyond amine oxidation, ketoABNO has also been applied to the oxidation of alcohols and aldehydes. More recently, its utility has extended beyond small-molecule transformations to include applications in protein modifications, such as serine-selective oxidative cleavage of proteins (in conjunction with a water-soluble copper-complex catalyst) and tryptophan-selective bioconjugation. In this Commentary, we highlight the development of ketoABNO as an oxidation catalyst and its emerging applications in biocompatible protein chemistry.

## Abstract 2

In celebration of our 15th anniversary and some of our most popular articles, Motomu Kanai reflects on the development of ketoABNO, following on from the report in 2012 in *Chemical Science* on catalysis mediated by ketoABNO (*Chem. Sci.*, 2012, https://doi.org/10.1039/C2SC20699D).

## Graphical abstract

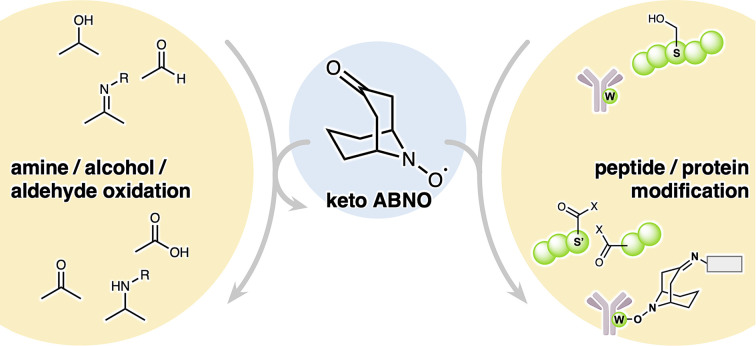

9-Azabicyclo[3.3.1]nonane-3-one *N*-oxyl (ketoABNO) was originally developed by Dupeyre and Rassa as a stable radical that persists at ambient temperature.^1^ Later, Iwabuchi and co-workers demonstrated that sterically less-hindered *N*-oxyl radicals—such as AZADO and ABNO, stabilized by Bredt’s rule—could broaden the substrate scope of TEMPO-catalyzed alcohol oxidation.^2^ Building on these foundational studies, we reported in *Chemical Science* the implementation of ketoABNO as a catalyst for the aerobic oxidation of amines in conjunction with a CuBr-ligand complex (https://doi.org/10.1039/C2SC20699D).^3^ This study established a highly efficient method for the aerobic oxidation of amines to imines under mild conditions (Fig. 1a). Furthermore, by employing a chiral copper–bisoxazoline complex, catalytic asymmetric amino acid synthesis was achieved from a glycine derivative and a nitroalkane (Fig. 1b). At the time, although *N*-oxyl radicals such as TEMPO were gaining attraction as oxidation catalysts,^4^ ketoABNO itself had received relatively little attention. While Kerton and co-workers independently reported CuBr_2_/TEMPO-catalyzed aerobic oxidation of amines around the same time (Fig. 1c),^5^ the ketoABNO system featured broader substrate scope and practical scalability. To support further studies, we developed a concise, three-step synthesis of ketoABNO, enabling gram-scale preparation (Fig. 1d). These features contributed to the growing recognition of ketoABNO, which, over the past decade, has emerged as a powerful catalyst/reagent for both alcohol oxidation and selective modification of amino acid residues in proteins. In this Commentary, we reflect on these developments and consider future directions.

**Fig. 1:**
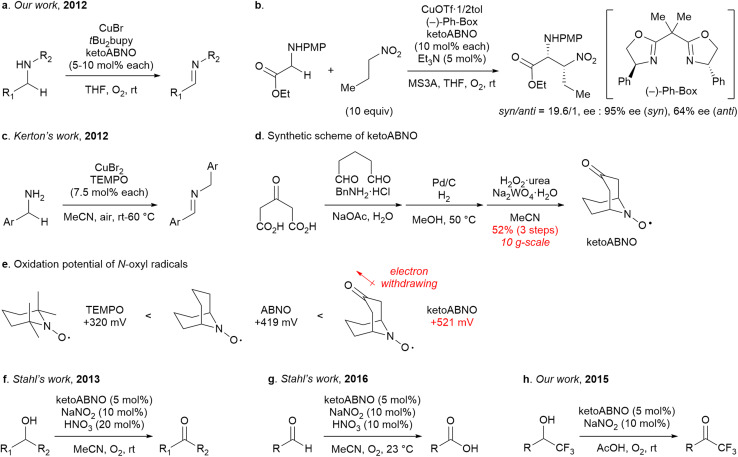
Utilities and properties of ketoABNO. (a) Catalytic aerobic oxidation of amines developed by our group. (b) Catalytic asymmetric oxidative coupling between amines and nitroalkanes. (c) Catalytic aerobic oxidation of amines by CuBr_2_/TEMPO catalysis developed by Kerton’s group. (d) Synthetic route of ketoABNO. (e) Oxidation potentials of *N*-oxyl radicals (*vs.* Ag/Ag^+^). (f) Catalytic aerobic oxidation of alcohols developed by Stahl’s group. (g) Catalytic aerobic oxidation of aldehydes developed by Stahl’s group. (h) Catalytic aerobic oxidation of fluoroalkyl alcohols developed by our group.

KetoABNO offers several advantages for oxidative transformations, including a high redox potential (+521 mV *vs.* Ag/Ag^+^) relative to TEMPO (+320 mV) and ABNO (+419 mV), as well as reduced steric hindrance (Fig. 1e). These properties enable selective and efficient aerobic oxidation of amines and alcohols in conjunction with copper complexes. For example, Zhang and co-workers employed a Cu salt/ketoABNO catalytic system in a micro-packed bed reactor for the aerobic oxidation of ethyl lactate to ethyl pyruvate, a valuable intermediate for pharmaceuticals and nutritional supplements.^6^ Notably, this system outperformed conventional Cu salt/TEMPO systems in both activity and selectivity.

Organocatalytic aerobic alcohol oxidation conditions without the use of metal-complex catalysts were developed by Liang and Hu,^7^ Iwabuchi,^2*b*^ and Stahl,^8^ who introduced NaNO_2_/O_2_-based cocatalytic systems for the reoxidation of *N*-oxyl radicals. These advances were subsequently adapted to ketoABNO systems (Fig. 1f), enabling metal-free aerobic oxidations. A key benefit of this advancement was the expanded substrate scope to include aldehydes, whose oxidation had previously been hindered by the acidity of the resulting carboxylic acids (Fig. 1g).^9^ Taking advantage of the high oxidation potential of ketoABNO, we later demonstrated the aerobic organocatalytic oxidation of α-fluoroalkyl alcohols to the corresponding fluoroalkyl ketones at room temperature (Fig. 1h).^10^ Additional modifications, such as the use of bismuth nitrate as an NO_*x*_ mediator, were introduced by Li and co-workers.^11^ Collectively, these contributions have firmly established ketoABNO as a versatile reagent for mild alcohol oxidation. Nevertheless, for many alcohol oxidations, ABNO remains a practical alternative due to its comparable reactivity and broader synthetic accessibility, including its frequent use as a precursor to functionalized ABNO derivatives.^12^

In parallel with advances in the small-molecule-directed methodology, ketoABNO has also found unique applications in biomolecular chemistry. Owing to its mildness and aqueous compatibility—critical features for protein chemistry—ketoABNO has enabled amino acid residue-selective chemical modifications of peptides and proteins. We demonstrated an artificial peptidase-type reaction—the serine-selective aerobic cleavage of peptide bonds—using a CuI/ketoABNO system, initiated by oxidation of the primary alcohol in the serine side chain (Fig. 2a).^13^ Oxidation of the hydroxymethyl group at serine to an aldehyde triggers hydrolysis of the peptide backbone. This reaction was applicable to proteins, with no detectable cleavage observed at threonine residues.

**Fig. 2:**
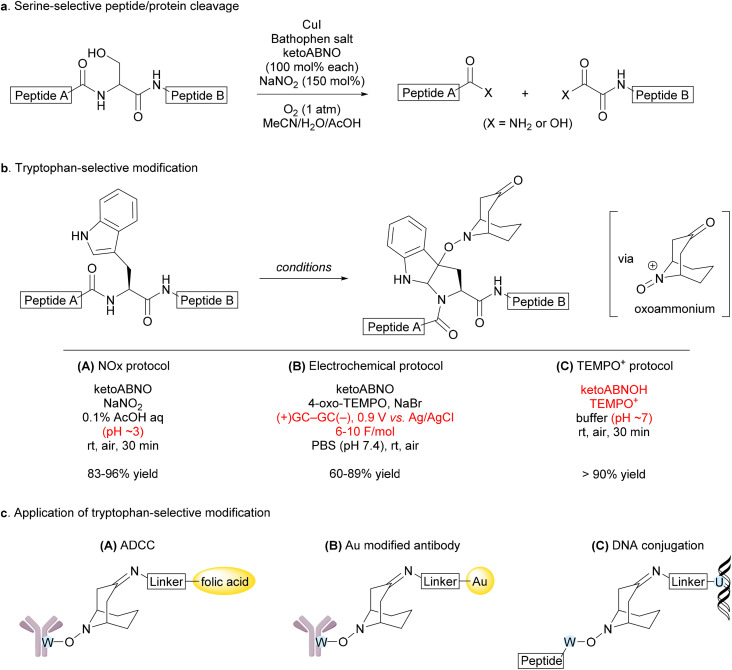
Applications of ketoABNO to peptide and protein modifications. (a) Serine-selective peptide/protein cleavage. (b) Tryptophan-selective bioconjugation. (c) Applications to life sciences.

During these studies, we serendipitously discovered that ketoABNO selectively forms an adduct with tryptophan residues, marking the first example of heavy-metal-free, tryptophan-selective protein modification.^14^ Under ketoABNO/NaNO_2_ conditions—without the use of a copper salt—in an acetonitrile/water/acetic acid mixture, the adduct was formed at the 3-position of tryptophan’s indole ring in peptides and proteins (Fig. 2b(A)). The reaction proceeded *via* a Friedel–Crafts mechanism with a ketoABNO-derived oxoammonium intermediate. Building on this mechanistic insight, we developed milder methods for generating the reactive oxoammonium species, such as electrochemical oxidation (Fig. 2b(B))^15^ and a ketoABNOH/TEMPO^+^ system (Fig. 2b(C)).^16^ The precise reason for the high chemoselectivity of ketoABNO-derived oxoammonium toward indoles remains to be elucidated.

Because tryptophan is the rarest of the canonical amino acids and is typically buried within proteins due to its hydrophobic characteristics, this method offers potential advantages for producing bioconjugates with high homogeneity.^17^ The ketone moiety of ketoABNO serves as a versatile handle for straightforward derivatization, enabling attachment to cytotoxic agents for constructing antibody–drug conjugates (ADCs),^18^ gold nanoparticles for the construction of antibody-derived cryo-electron microscopy probes,^16^ and nucleic acids for generating DNA–protein conjugates^19^ (Fig. 2c). This synthetic methodology holds promise for broader application in the development of novel biological tools, diagnostic agents, and therapeutics.

In summary, we have outlined the evolution of ketoABNO chemistry since our initial report in 2012. The reagent’s ease of use—requiring only simple mixing with a cocatalyst and/or oxidant, including molecular oxygen—combined with its water tolerance, mild and tunable reactivity, and high chemoselectivity, makes it a highly attractive tool in modern synthetic chemistry and pharmaceutical sciences. We anticipate continued growth of this platform at the interface of synthetic methodology, biology, and medicine.

## Author contributions

M.T. and M.K. wrote the manuscript.

## Conflicts of interest

There are no conflicts to declare.

## Data Availability

There is no additional data associated with this article.
